# Malignant hyperthermia in Poland: A survey study

**DOI:** 10.1097/MD.0000000000033238

**Published:** 2023-03-10

**Authors:** Agnieszka Cieniewicz, Janusz Trzebicki

**Affiliations:** a 1st Department of Anesthesiology and Intensive Care, Medical University of Warsaw, Warsaw, Poland.

**Keywords:** anesthesia, dantrolene, malignant hyperthermia, perioperative complications

## Abstract

Malignant hyperthermia (MH) is a life-threatening syndrome caused by sudden, uncontrolled skeletal muscle hypermetabolism in response to inhalation anesthetics and depolarizing relaxants. The estimated incidence of MH is between 1:10,000 and 1:250,000 anesthetic procedures. In Poland, due to lack of reporting, the incidence of MH is unknown. Dantrolene is imported as a life-saving drug (target import) and temporally authorized for sale. The aim of the study was to evaluate the prevalence of malignant hyperthermia in Poland and to assess the accessibility to dantrolene in Poland. A questionnaire was conducted among the chiefs of anesthesia and intensive care units in Poland. During the years 2014 to 2019, 10 episodes of MH have been reported in 238 surveyed polish anesthesia departments. The estimated prevalence is 1:350,000. Eight patients survived the MH crisis. Dantrolene is stocked in 48 (20%) anesthesiology departments. Among the surveyed hospitals, only in 38 (16%) it is possible to administer dantrolene within 5 minutes of suspecting a MH reaction. Less than half units (44%) have an algorithm for the management of MH episode in the operating theaters. The results of the study revealed, that the prevalence of MH in Poland is lower than the prevalence reported in other countries. Access to dantrolene in Poland is limited.

## 1. Introduction

Malignant hyperthermia (MH) is a life-threatening syndrome caused by sudden skeletal muscle hypermetabolism in response to inhalation anesthetics and depolarizing muscle relaxants. The estimated prevalence of MH is between 1:10,000 and 1:250,000 anesthetic procedures.^[1–4]^ In 2004 Mayzner-Zawadzka reported that 10 cases of MH occurred annually in Poland.^[5]^ Nowadays, due to lack of a central database and mandatory reporting, the incidence is unknown. There is no MH unit in Poland that provides in vitro contracture testing (IVCT) and genetic testing is not reimbursed. Dantrolene is the most important element of causal treatment of an MH reaction, proved to reduce mortality.^[1,6]^ However, it is unknown how many polishes hospitals stock and have access to dantrolene. No dantrolene availability database exists in Poland.

According to the data of Statistics Poland, in 2019 there were 890 general hospitals in Poland with 480 anesthesiology and intensive care units and 3537 operating rooms. In 2019 more than 2 and a half million operations were performed, of which over 1 million 4 hundred thousand under general anesthesia.^[7]^ In Poland, dantrolene is imported as a life-saving drug (target import) and temporally authorized for sale (no permission based on the Regulation of the Minister of Health of 2001 is required).^[8]^ It is expensive and not reimbursed by the National Health Fund.

## 2. Objectives

The main objective of this study was to evaluate the prevalence of malignant hyperthermia in Poland using a questionnaire sent to chiefs of anesthesia departments. The secondary objective was to assess the accessibility to dantrolene in Poland.

## 3. Material and methods

A questionnaire was created in Google Forms and a link e-mailed to the voivodship consultants of anesthesia and intensive care after prior contact by phone. They were asked to forward the questionnaire to the chiefs of anesthesia departments in their voivodships. In all, questionnaires were sent to 453 departments in 16 voivodships. We reached 94% of all polish anesthesia and intensive care departments. We first contacted the consultants in August 2020. In January and November 2021, we sent reminders, asking to distribute the questionnaires once again. The survey electronic link was accessible from August 1, 2020 to December 31,2021.

The questionnaire consisted of 17 questions and could be completed within 5 minutes. We asked how many MH episodes occurred in the past, with emphasis on the last 5 years – 2014 to 2019, their treatment, access to dantrolene and to a MH crisis algorithm. The survey included also questions about the respondent’s department, such as number of operating rooms, population treated, drugs used for anesthesia. Respondents were asked to provide the name of the department, but it was not mandatory. No personal data were collected. A polish-language version of the questionnaire was used. The complete questionnaire translated into English language is shown in Table 1. On completion of the survey, the results were imported as a data in a spreadsheet.

**Table 1:** Complete questionnaire.

Name of the hospital
Population treated
Children
Adults
Adults and children
Number of operating rooms
Number of ICU beds
In your hospital, which malignant hyperthermia triggers are used?
Succinylcholine
Volatile anesthetics
Is a malignant hyperthermia algorithm available in the operating rooms?
Has a malignant hyperthermia crisis ever occurred in the hospital? If yes, how many episodes?
How many malignant hyperthermia episodes have occurred during the years 2014–2019?
Did the patient receive dantrolene?
Did the patient survive the malignant hyperthermia crisis?
Is dantrolene stocked in your hospital?
If no, do you know where is it stocked?
How fast is it possible to administer dantrolene in case of a malignant hyperthermia crisis?
<5 min
5–60 min
>60 min
I don’t know
Was patient referred to malignant hyperthermia diagnostic unit?
If yes, to which center?
What kind of diagnostic was performed?
City population
<100,000 habitants
100,000–500,000 habitants
>500,000 habitants

Our study was approved by the Bioethical Committee of the Medical University of Warsaw (AKBE/256/2019) on June 10, 2019.

## 4. Results

Questionnaires were returned from 238 departments, which constitutes a response rate of 53%. All responses were eligible for further analysis. Overall, this corresponds to 1638 operating rooms. In 143 (60%) departments only adults were treated, 12 (5%) were pediatric hospitals. Of the responding departments, in 83 (35%) both populations were treated. Only 5 from 238 anesthesia departments do not use volatile anesthetics, while 23 do not use succinylcholine.

Our study showed, that during the years 2014 to 2019, 10 suspected cases of MH were reported in 238 surveyed polish anesthesia departments. Assuming that each year 1 million four hundred thousand operations under general anesthesia are performed in 480 departments, therefore 7 million in 5 years and taking into consideration that we obtained data from half of polish anesthesia departments, we may assume that 20 suspected cases of MH may have occurred in Poland during this period. This indicates the prevalence of 1:350,000. Two out of 10 patients did not survive the suspected MH crisis. In 1 case the patient did not receive dantrolene. Of the surviving patients, 6 were referred for further diagnostics: 3 for genetic testing, one for the IVCT and 2 for both. In 2 cases, the head of the department completing the questionnaire did not know whether further investigation had been performed.

Dantrolene is stocked in 20% (n = 48) of the responding polish departments of anesthesiology and intensive care: in 14 adult departments, 9 pediatric departments and 25 where both populations were treated. Dantrolene is stocked most often in medium-sized cities (population between 100,000 and 500,000). Among departments where dantrolene is stocked, only 38 are able to administer it within 5 minutes of diagnosis of a suspected MH reaction. In 10 departments possessing a stock of dantrolene and an additional 70 departments with access from a neighboring medical facility, it was possible to administer dantrolene within 60 minutes (n = 80). Ninety-eight chiefs of anesthesia’s department declared, that they have access to dantrolene in a time longer than 60 minutes. Of the responding departments, 22 did not have knowledge how fast they can get dantrolene in case of an MH crisis. Algorithm for the management of MH episode is available in the operating rooms in 104 units (44%). All the above data are collected in Tables 2 and 3.

**Table 2:** Availability of dantrolene in 238 polish hospitals.

Is dantrolene stocked in your hospital?	
Yes	48 (20%)
No	190 (80%)
If no, do you know where is it stocked?	
Yes	170
No	20
How fast is it possible to administer dantrolene in case of a malignant hyperthermia crisis?	
<5 min	38 (16%)
5–60 min	80 (34%)
>60 min	98 (41%)
I don’t know	22 (9%)
Dantrolene in stocked in hospitals located in cities with population of:	
<100,000 habitants	10/122
100,000–500,000 habitants	21/54
>500,000 habitants	17/62

**Table 3:** Detailed results of the questionnaire.

Results	Number of hospitals	Number of responses	MH episodes (2014–2019)	MH deaths (2014–2019)	Number of hospitals stocking dantrolene	Number of hospitals that are able to administer dantrolene within				MH algorithm in operating rooms*
Voivodeship						5 min	5–60 min	>60 min	Don’t know	
Dolnoslaskie	29	14	1	0	8	8	5	1	0	5
Kujawsko-Pomorskie	25	12	0	0	2	1	4	4	3	5
Lubelskie	25	11	1	1	3	1	4	6	0	3
Lubuskie	13	12	0	0	1	1	2	8	1	1
Lodzkie	33	16	0	0	5	5	3	6	2	8
Malopolskie	27	10	0	0	1	0	6	3	1	5
Mazowieckie	83	60	4	1	11	6	21	28	5	23
Opolskie	8	1	1	0	1	1	0	0	0	1
Podkarpackie	27	6	0	0	0	0	2	3	1	3
Podlaskie	24	22	0	0	3	3	11	8	0	18
Pomorskie	16	11	1	0	1	1	4	6	0	4
Slaskie	42	15	0	0	4	4	4	6	1	8
Swietokrzyskie	20	8	0	0	2	2	2	3	1	4
Warminsko-Mazurskie	20	12	1	0	2	1	5	5	1	5
Wielkopolskie	40	16	1	0	2	2	5	7	2	7
Zachodniopomorskie	21	12	0	0	2	2	2	4	4	4
Poland	453	238	10	2	48	38	80	98	22	104

MH = malignant hyperthermia.

* Number of hospitals where an algorithm for the management of MH crisis is available in the operating rooms.

## 5. Discussion

Differences in prevalence of MH have been reported in previous studies.^[2–4]^ However, there is no data regarding MH prevalence in Poland. A study regarding Mazovia voivodeship has been published in 2022, but no nationwide study has been conducted.^[9]^ This is the first study regarding this topic.

Our study showed, that the estimated prevalence of MH due to anesthesia is around 1:350,000. It is lower than reported in previously published studies, for example, in New York (1:100,000).^[2–4]^ In contrast to our study, the other authors obtained the data from national official registers, which are not available in Poland regarding perioperative events. Therefore, lower quality of the data needs to be considered.

In the 1970s, reported fatality rate was 64%.^[10]^ In our study, the fatality rate was 20%. Thanks to dantrolene and advances in intraoperative monitoring techniques, the fatality rate has significantly decreased. Based on the data by Larach et al^[6]^, fatality rate in North America was assessed to be 1.4%. In Japan, the fatality rate was reduced from 40% to 5.9% over 4 decades.^[3]^ In our study, one of the patients who did not survive the MH crisis did not receive dantrolene.

A possible explanation for the high fatality rate in Poland is the low availability of dantrolene. According to our findings, only 20% of polish anesthesia departments stock dantrolene. Interesting to note is that only 38 of these departments, what comprised 16% of all polish hospitals, are able to administer dantrolene within 5 minutes of a diagnosis of a suspected MH crisis, whereas the European malignant hyperthermia group (EMHG) recommends that it should be attainable in every institution where MH trigger drugs are used.^[11]^ Previous studies have shown that delay in administering dantrolene increases the likelihood of complications.^[12,13]^ According to our data, only 80 more units can give dantrolene within 60 minutes. Half of the hospitals are not able to provide dantrolene in 1 hour. Therefore, the majority of patients treated in these hospitals are at risk of complication of MH and will develop them. In our opinion, this is one of the most important findings from the survey.

A reason for low availability of dantrolene may be its price. The drug is relatively expensive (4433 PLN for 12 vials) and is not reimbursed by the National Health Fund. EMHG recommends that typical stock level of 36 vials of dantrolene should be immediately available. However, if further dantrolene cannot be obtained within 30 minutes a stock level of 48 vials is recommended and if further dantrolene cannot be obtained within 1 hour, EMHG recommends a stock level of 60 vials.^[11]^ This can constitute an additional financial challenge for hospitals.

Of the surviving patients, only 6 were referred for further diagnostics. The gold standard for predictive testing for malignant hyperthermia is the IVCT. In Poland an MH unit has never been established and the IVCT is not available. Muscle biopsy screening can be done only abroad. MH susceptibility diagnosis can be also based on DNA screening, which can be performed in Poland. However, this genetic analysis is not reimbursed and the cost is relatively high (2000 PLN). In view of the fact that this was not the objective of our survey, we do not know the results of surviving patients’ tests and whether family members of the survivors have been referred for further diagnostics.

Patient safety is a priority. We all hope that a MH reaction will not happen to us but if it does, we need to have knowledge about its management. The Helsinki Declaration on Patient Safety in Anesthesiology states that institutions providing perioperative anesthesia care should have protocol for managing MH.^[14]^ An algorithm for the management of MH crisis is available only in half of the operating rooms of polish anesthesia departments. Implementation of emergency manuals improves patient care during critical events.^[15]^

## 6. Limitations of the study

This study has some limitations that should be discussed. The results of the survey need to be interpreted with caution as the data were obtained from chiefs of the departments, not medical database. Because it was retrospective, some information may have been missed or inaccurate. The actual occurrence of MH may be underestimated also due to non-responder bias. A possible limitation of the survey may be the response rate of 53%. On the other hand, it is higher than an average response rate of about 38% for surveys involving healthcare providers.^[16]^ We also consider the data obtained from all voivodeships as a strong point of the questionnaire. The questionnaire was distributed by e-mail and accessed by link, so it could be theoretically accessed multiple times by the same respondent. Because of the theme of the survey, it seems unlikely. Accepting these limitations, the findings deserve attention.

## 7. Conclusions

It is the first study estimating MH prevalence in Poland. It showed, that it is lower than the prevalence reported in other countries. But to have a full understanding of MH prevalence in Poland, a national register should be created. Nevertheless, our study revealed, that access to dantrolene, especially with no time delay, is limited. It shows an immediate need for action to improve patients safety by creating a net of dantrolene distribution.

## Acknowledgments

We thank all the voivodship consultants for their logistical support. We also thank the national consultant of anesthesia and intensive care, prof Radoslaw Owczuk, for his endorsement of this study.

## Author contributions

**Conceptualization:** Agnieszka Cieniewicz, Janusz Trzebicki.

**Data curation:** Agnieszka Cieniewicz, Janusz Trzebicki.

**Formal analysis:** Agnieszka Cieniewicz, Janusz Trzebicki.

**Investigation:** Agnieszka Cieniewicz, Janusz Trzebicki.

**Methodology:** Agnieszka Cieniewicz, Janusz Trzebicki.

**Project administration:** Agnieszka Cieniewicz, Janusz Trzebicki.

**Resources:** Agnieszka Cieniewicz, Janusz Trzebicki.

**Supervision:** Agnieszka Cieniewicz, Janusz Trzebicki.

**Validation:** Agnieszka Cieniewicz, Janusz Trzebicki.

**Writing – original draft:** Agnieszka Cieniewicz, Janusz Trzebicki.

**Writing – review & editing:** Agnieszka Cieniewicz, Janusz Trzebicki.
